# Neuroprotective pentapeptide CN-105 is associated with reduced sterile inflammation and improved functional outcomes in a traumatic brain injury murine model

**DOI:** 10.1038/srep46461

**Published:** 2017-04-21

**Authors:** Daniel T. Laskowitz, Haichen Wang, Tony Chen, David T. Lubkin, Viviana Cantillana, Tian Ming Tu, Dawn Kernagis, Guanen Zhou, Gary Macy, Bradley J. Kolls, Hana N. Dawson

**Affiliations:** 1 Department of Neurology, Duke University School of Medicine, Durham, NC 27710, USA; 2 Department of Neurobiology, Duke University School of Medicine, Durham, NC 27710, USA; 3 Aegis-CN LLC., Durham, NC, USA; 4 Department of Neurology, National Neuroscience Institute, Tan Tock Seng Hospital, Singapore

## Abstract

At present, there are no proven pharmacological treatments demonstrated to improve long term functional outcomes following traumatic brain injury(TBI). In the setting of non-penetrating TBI, sterile brain inflammatory responses are associated with the development of cerebral edema, intracranial hypertension, and secondary neuronal injury. There is increasing evidence that endogenous apolipoprotein E(apoE) modifies the neuroinflammatory response through its role in downregulating glial activation, however, the intact apoE holoprotein does not cross the blood-brain barrier due to its size. To address this limitation, we developed a small 5 amino acid apoE mimetic peptide(CN-105) that mimics the polar face of the apoE helical domain involved in receptor interactions. The goal of this study was to investigate the therapeutic potential of CN-105 in a murine model of closed head injury. Treatment with CN-105 was associated with a durable improvement in functional outcomes as assessed by Rotarod and Morris Water Maze and a reduction in positive Fluoro-Jade B stained injured neurons and microglial activation. Administration of CN-105 was also associated with reduction in mRNA expression of a subset of inflammatory and immune-related genes.

Traumatic brain injury(TBI) remains a leading cause of mortality in the United States, accounting for approximately one third of injury-related deaths and with an annual prevalence of approximately 1.7 million patients^1^,^2^. Long-term neurological morbidity following TBI is also common, and almost one half of all patients who are hospitalized with severe TBI will be left with significant neurocognitive sequelae that may impair quality of life^3^,^4^. Unfortunately, therapeutic options for patients with TBI remain limited and are primarily focused on treating intracranial hypertension, optimizing cerebral perfusion, and providing supportive care.

One important mechanism that may contribute to progressive cerebral edema and delayed neuronal injury is the sterile neuroinflammatory response. Acute CNS injury triggers immune activation in the absence of microbial infection through the release of damage-associated molecular pattern molecules(DAMPs), which can have both adaptive and maladapative effects on recovery^5^. Activation of microglia and recruitment of peripheral mononuclear cells into the CNS compartment represents the cornerstone of the CNS neuroinflammatory response; both are associated with the release of inflammatory cytokines. This inflammatory cascade contributes to oxidative stress, secondary neuronal injury, blood brain barrier break down, and resulting cerebral edema. Although a variety of immunomodulatory strategies, including the use of glucocorticoids^6^ have been tried in the setting of acute brain injury, there are no pharmacological interventions that have been associated with an improvement in long term functional outcomes^7^.

It is increasingly recognized that genetic influences play an important role in modifying acute brain injury responses and outcome after TBI. One of the most robust genetic associations with outcome after TBI is the apolipoprotein E(APOE - gene; apoE - protein) polymorphism. There are three distinct apoE protein isoforms, designated apoE2, apoE3, and apoE4. Although there are conflicting reports on the effect of APOE4 and poor prognosis after subarachnoid hemorrhage^8^,^9^ and intracranial hemorrhage^10^,^11^, cumulative evidence suggests that APOE4 is associated with poor outcome after acute brain injury, and in particular following traumatic brain injury^12^,^13^,^14^,^15^,^16^,^17^. These data underscore the importance that apoE plays in mediating CNS responses to acute injury and neurodegeneration.

The apoE protein was initially found to reduce glial activation and inflammatory cytokine release *in vitro*^18^, and these results were extended to our murine model of TBI, where the absence of endogenous apoE exacerbated post-injury neuroinflammation and development of cerebral edema^19^. However, the intact apoE holoprotein does not readily cross the blood brain barrier(BBB), and would thus be unsuitable for peripheral administration^20^. To address this problem, we originally created a series of apoE mimetic peptides derived from the apoE receptor binding region(residues 130–150)^21^,^22^. These peptides had robust efficacy in cell culture^22^ and improved functional and histological endpoints in preclinical models of CNS inflammation^23^ and acute brain injury associated with many of the pathological features of clinical TBI, including intracranial^24^ and subarachnoid hemorrhage^25^,^26^ and both closed skull and cortical contusion models of brain trauma^27^,^28^,^29^,^30^.

In the current study, we developed a smaller peptide(CN-105) that was designed by modeling the polar receptor binding face of the helical apoE receptor binding region(Ac-VSRRR-amide). We now test the hypothesis that intravenous administration of CN-105 dampens neuroinflammatory responses and thus possibly improves functional outcomes in a murine model of closed head injury. Moreover, we address variables that may inform the translation of CN-105 to the clinical setting, including defining the optimal dosing paradigm and latency from injury in which treatment continues to exert neuroprotective effects. Since microglial activation and subsequent neuroinflammation via specific receptor interactions may be one mechanism by which apoE modifies outcome following CNS injury^22^,^23^,^30^,^31^,^32^,^33^,^34^, we therefore also examined possible mechanisms of action by assessing histology and inflammatory gene expression.

## Results

### CN-105 is associated with improved behavioral function

**Figure 1:**
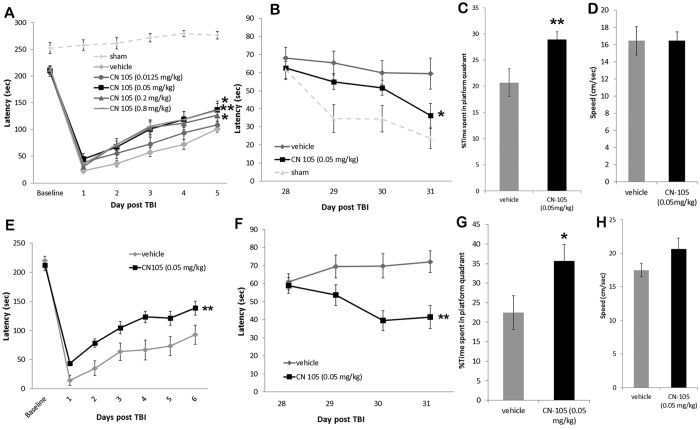
CN-105 improves vestibulomor and cognitive function. Following TBI, intravenous administration of 0.05 mg/kg of CN-105 was the lowest effective dose resulting in improved vestibulomotor functional performance, as assessed by Rotarod(**A**) and cognitive function as assessed by the Morris Water Maze(**B**) and the MWM probe trial(**C**). There was no difference in swim speed on the MWM(**D**). CN-105(0.05 mg/kg) administered 4 hours post-injury was also associated with a significant and durable improvement in Rotarod latency(**E**) and by reduced deficit on MWM(**F**) and the MWM probe trial(**G**) No significant difference in speed was detected between the two treatment groups(**H**). For comparison purposes results from sham mice are shown(dotted lines) in panels A and B but are not included in statistical calculations. Asterisks denote significant differences in performance as measured by ANOVA; *p < 0.05 and ** p < 0.01.

The murine closed head injury model used in this study creates a diffuse injury which presents with vestibulomotor and cognitive dysfunction, neuroinflammation and a degenerating neuronal population the hippocampus and cortex^19^,^35^. This model is designed to recapitulate the diffuse glial activation, cerebral edema, and injury to preferentially vulnerable areas of cortex and hippocampus that result in vestibulomotor dysfunction and persistent neurocognitive deficits associated with moderate clinical traumatic brain injury. To establish a dose response for CN-105 treatment following acute brain injury, we compared a range of dosing concentrations with vehicle following TBI. Two hours after injury, CN-105 was administered intravenously at doses of 0.0125, 0.05, 0.2, or 0.8 mg/kg or vehicle(n = 11–12/group) and animals were subsequently tested for functional performance. We assessed vestibulomotor performance on the Rotarod, and compared the CN-105 treated mice to vehicle treated mice. When compared to vehicle, improved functional performance by Rotarod was demonstrated with the 0.05, 0.2 and 0.8 mg/kg doses(p = 0.011, 0.023 and 0.009, respectively as assessed by two level ANOVA) while the 0.0125 mg/kg dose did not show robust improvement(p = 0.267)(Fig. 1A). The Rotarod performance of sham injured controls is shown for illustrative comparison only and was not included in the statistical analysis(Fig. 1A). Thus, our dose-response results demonstrated that 0.05 mg/kg CN-105 was the lowest effective dose resulting in improved functional performance, and this dose was used in subsequent experiments.

Since neurocognitive impairment is a common aftermath of TBI in the clinical setting and in our model, mice receiving the 0.05 mg/kg dose also received long term functional assessment of spatial learning and memory at 28 days post-injury by quantifying Morris Water Maze latency^36^. CN-105 treated mice significantly reduced time to escape to the platform compared to vehicle treatment(p = 0.036)(Fig. 1B), suggesting a reduction in cognitive impairment following injury. The Morris Water Maze performance of sham injured controls is shown for illustrative comparison only and was not included in the statistical analysis(Fig. 1B). Furthermore, the CN-105 treated group performed better on the probe trial(Fig. 1C) confirming the treatment better preserved memory(p = 0.0079) than treatment with vehicle. There was no difference in swim speed(Fig. 1D) suggesting motor differences were minimal between the two groups(p = 0.4968) at this time point.

After establishing dose response, we next determined if we could extend the therapeutic window of CN-105 administration following TBI in this murine model. The lowest effective dose(0.05 mg/kg) of CN-105 administered at 4 hours post-injury resulted in a significant and durable improvement in Rotarod latency as compared to vehicle(Fig. 1E). This dosing paradigm also conferred long-term improvement in neurocognitive performance as quantified by a reduced deficit on Morris Water Maze testing(Fig. 1F). Furthermore, the CN-105 treated mice performed better on the probe trial(Fig. 1G) confirming the treatment better preserved memory(p = 0.0211) than treatment with vehicle. There was no significant difference in swim speed(Fig.1H) suggesting motor differences were minimal between the two groups at this time point(p = 0.0567). Pretreatment of mice 30 minutes prior to TBI had no additional effects on vestibulomotor and cognitive function(data not shown).

### CN-105 is associated with reduced microgliosis and neuronal injury following TBI

**Figure 2:**
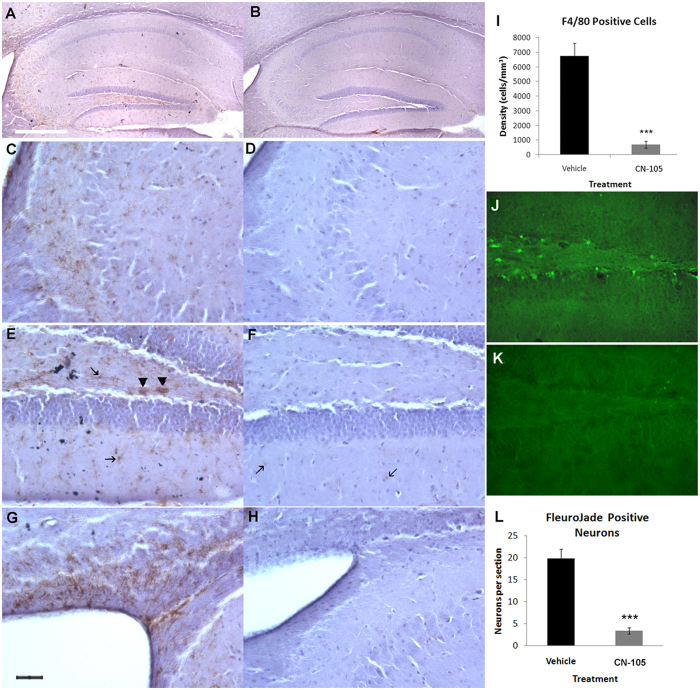
CN-105 reduces microgliosis and neuronal injury. Comparison images of activated F4/80 immunostained microglial in the hippocampus 10 days post-TBI, brain slices from vehicle(**A**,**C**,**E**,**G**) and CN-105(**B**,**D**,**F**,**H**) treated mice, unbiased stereology confirms that treatment with CN-105 is associated with a reduction in microgliosis(p = 0.0002, ***)(**I**). Higher powered images of microglia in the CA3 region(**C**,**D**) in the polymorphic region(**E**,**F**) and in corpus callosum and fimbria of the periventricular region(**G**,**H**). Microglia are indicated in with arrows and phagocytic microglia with arrowheads in **E** and **F**. Images C-H are at 20x magnification and the black bar in G represents 40 μm. Images A and B are at 4x magnification and the white bar in A represents 250 μm. Comparison images of FJB-stained brain slices 24 hours post-TBI show degenerating neurons in the hilus of the dorsal dentate gyrus of TBI vehicle treated mice(**J**) which are significantly reduced by treatment with CN-105(**K**), quantification p < 0.0001(**L**).

Since microglial activation and subsequent neuroinflammation via specific receptor interactions may be one mechanism by which apoE modifies outcome following CNS injury^22^,^23^,^30^,^31^,^32^,^33^,^34^ we assessed differences in microglial activation between 0.05 mg/kg CN-105 treated and vehicle treated animals 10 days post TBI. Although microgliosis is also prominent in the cortex of closed head injury models, quantification of hippocampal inflammation is an important histological endpoint as it tends to be less variable than injury directly underlying the mechanical injury. A high level of microgliosis, as characterized by F4/80 immunostaining, is clearly evident in the hippocampus of the TBI injured mice(Fig. 2A), especially in the CA3 region(Fig. 2C), in the hilus of the dentate gyrus(Fig. 2E) and in the corpus callosum and fimbria surrounding the lateral ventricle(Fig. 2G). Treatment with CN-105 greatly reduced F4/80 immunopositive microglia in TBI injured mice as seen by microscopy(Fig. 2B,D, F and H). Quantification of F4/80 microglia in the dorsal hippocampus of TBI injured mice using unbiased stereology techniques showed a significant decrease in density of microglia in CN-105 treated mice compared to vehicle treated mice(Fig. 2I). Furthermore, microglia in the vehicle treated group displayed hypertrophy in the form of increased cytoplasmic volumes and thickness of processes indicative of microglial activation(Fig. 2C,E and G). Microglia from the CN-105 treated group(Fig. 2D,F and H) were almost exclusively small ramified microglia indicative of resting microglia with small cell bodies and thin, barely visible processes. Bushy, amoeboid, microglia that are associated with the phagocytic form were present throughout the hippocampus in the vehicle treated group only(Fig. 2E, arrowheads).

Degenerating neurons in the dentate gyrus have been previously reported and there is evidence that doublecortin-expressing late neural progenitors are relatively vulnerable to brain injury^37^. Differences in neuronal injury between 0.05 mg/kg CN-105 treated and vehicle treated animals were assessed 24 hours post-injury by Fluoro-Jade B(FJB) staining. Significantly fewer Fluoro-Jade B positive degenerating neurons were present in the hilus of the dentate gyrus of CN-105 treated mice compared to vehicle treated mice(Fig. 2J and K). A number of vehicle treated mice also showed FJB staining in the CA3 region of the hippocampus, however, staining in the dentate gyrus is more consistent and was therefore used for quantification. Counting of the FJB positive neurons in every eighth brain slice of the dorsal hippocampus confirmed a significant decrease in the number of Fluoro-Jade B positive neurons(p < 0.00001)(Fig. 2L). Some of the FJB positive cells are located in the subgranular zone where adult neurogenesis occurs and may represent doublecortin(DCX)-expressing late neural progenitors. In sum, these results strongly suggest that administration of CN-105 is associated with a reduction of neuronal injury in the hippocampus.

### CN-105 is associated with changes in inflammatory gene expression patterns following TBI

To further evaluate the effect of CN-105 on neuroinflammatory pathways, we assessed gene expression using a pathway array specific for inflammatory and immune responses. Four cohorts of mice were sacrificed at 24 hours post-injury; sham plus vehicle treated(S-v) mice, sham plus CN-105 treated(S-CN) mice, TBI plus vehicle treated(TBI-v) mice, and TBI plus CN-105 treated(TBI-CN) mice. CN-105(0.05 mg/kg) and vehicle were administered 2 hours following injury. Relative gene expression based fold-change was calculated from raw threshold cycle data, using beta-actin and glyceraldehyde 3-phosphate dehydrogenase(GAPDH) as internal standards for normalization. We first established the effect of TBI on these inflammatory pathways by normalizing RNA levels from injured animals receiving vehicle to non-injured sham animals receiving vehicle(Table 1). As expected, there were changes in mRNA levels of a number of inflammatory and immune-related genes in response to the closed head injury. Of the 84 genes evaluated, 57 genes were upregulated, 12 genes were downregulated, and 10 genes were unchanged(Table 1).

Several of the most highly upregulated genes in this array 24 hours post TBI are members of the TLR signaling pathway, notably *Tlr6* and *Myd88*, and to a lesser extent *TLR4, TLR7, TLR1, Tirap, TLR2* and *Nfkb1*, the DNA binding unit of NF-κβ(Table 1). As expected the upregulation of the TLR pathway and activation of NF-κβ leads to the upregulation of a number of pro-inflammatory cytokine genes, including, *IL1α* and *β, IL-6, IL-22*, and *TNFα* and *TNFβ*. Also highly induced are the primary and secondary activators of macrophages, *INFγ* and the receptor for *CD40l, CD40*. Furthermore, chemokine genes such as *Cxcl3* also known as the growth-regulated protein(GRO), protein gamma and macrophage inflammatory protein-2-beta(MIP2b), *Ccl11*(eotaxin-1) and *Ccl20*(NIP3A), *Ccl24*(eotaxin-2), *Cxcl9*(MIG), *Cxcl1* and *Cxcl10*(Table 1) were also among the most upregulated genes following TBI. These chemokines control migration and adhesion of cells of the immune response such as monocytes, eosinophils, lymphocytes, and T-cells. Concurrently, the expression of the anti-inflammatory *IL-10* gene is highly increased after TBI. IL-10 expression is also activated through the TLR pathway. Interestingly IL-10 has been shown to play a role in autoimmune disease(reviewed in ref. 38). Of the 12 genes downregulated by TBI, *Nr3c1, Bcl6, IL18, Nfatc3* and *IL1r1* were some of most highly repressed genes. Of note is the robust downregulation of the *Bcl6* gene, which codes for a transcription repressor implicated in many cellular pathways to include inflammation.

**Figure 3:**
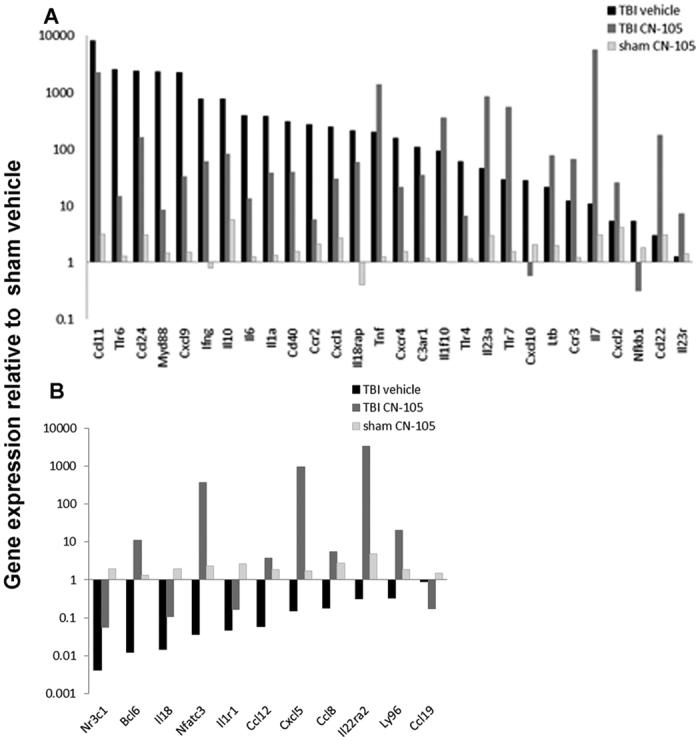
CN-105 ameliorates changes in inflammatory gene expression. One day post-injury, differential inflammatory gene expression analysis demonstrated changes in gene expression relative to sham vehicle treated controls for TBI CN-105 treated and untreated mice. Results are organized by upregulated(**A**) and downregulated(**B**) expression of genes from vehicle treated TBI mice relative to vehicle treated sham mice. Results from CN-105 treated TBI and sham mice relative to vehicle treated sham mice are also included. Only genes from TBI CN-105 treated mice that showed more than a 3 fold change when compared to TBI vehicle treated mice are presented.

After establishing the effect of TBI on inflammatory mRNA expression, we next assessed the effect of CN-105 following TBI(Tables 1 and 2; Fig. 3). Represented in Fig. 3 are genes from TBI-v brains that show a greater than 3 fold difference when compared to TBI-CN brains. Results of sham-v mice are also presented for comparison. The values for all 3 groups are shown relative to sham-v gene expression. Of the 57 genes that were upregulated by TBI, the overexpression of 28 genes was substantially(as defined by fold change >3) altered by treatment with CN-105(Fig. 3A). Of note, the post-injury upregulation of TLR pathway genes such as *Tlr6, Myd88, TLR4*, and the DNA binding unit of NF-κβ post TBI was reduced by treatment, as were the TBI upregulated cytokines *IL6, IL1α, IL10* and the primary activators of microglia/macrophages *INFγ* and *CD40*(Fig. 3A). Chemokine genes such as *Cxcl9, Cxcl10, Ccl24, Cxcl1* were also lower than in the vehicle treated TBI group, as were the genes coding for chemokine receptors *Ccr2* and *Cxcr4* and *IL18rap*, the accessory subunit of the heterodimeric receptor for IL18, required for the activation of NF-κB and JNK in response to IL18. The expression of several genes upregulated by TBI is upregulated to a higher level by treatment with CN-105(Fig. 3A). Several of these genes such as *Ccl22* and *Il-7* are associated with recovery and repair following injury. *IL-7* has been shown to promote neuronal survival, while *Ccl22* is associated with M2 macrophage polarization. Although the function of *IL1f10*(corresponds *to IL-38* in humans) is a recently discovered cytokine it has been shown to inhibit inflammatory responses^39^,^40^,^41^. TBI-associated downregulation of the *Bcl6* was significantly reversed, to above sham levels by CN-105(Fig. 3B). In aggregate, these results are consistent with the hypothesis that administration of CN-105, has the overall effect of downregulating neuroinflammatory responses following TBI.

To assess the effects of CN-105 on inflammatory gene expression on control mice, mRNA from sham CN-105 treated mice was compared to sham vehicle treated mice. Gene expression increase greater than 3 fold was observed in 13 genes(Table 1). No downregulation of gene expression was observed.

### CN-105 demonstrates penetration into the CNS compartment

**Figure 4:**
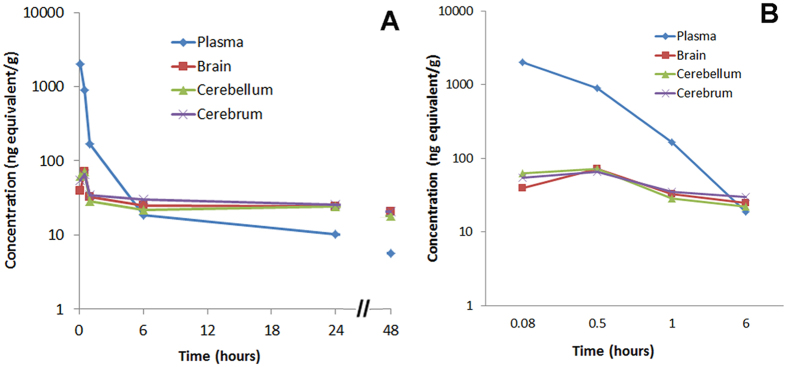
Pharmacokinetic studies of CN-105 demonstrate CNS bioavailability. Concentration of [^14^C]- radioactivity in the plasma and central nervous system of male CD-1 mice following an intravenous dose of [^14^C]-radiolabeled CN-105 peptide(**A**). For clarity, the concentration of [^14^C]- radioactivity during the first 6 hours only is depicted in(**B**).

One rationale for creating the smaller apoE mimetic peptides was to enhance CNS penetration and possibility of noninvasive mechanisms of delivery(for example, intranasal administration). Of note, a higher dose of CN-105 and different mouse strain was utilized in these outsourced experiments(CD-1 vs. C57-BL/6), although prior reports suggest that this would not affect pharmacokinetic results^42^,^43^. To define pharmacokinetic parameters, we performed pharmacokinetic studies of CN-105 following a single 1.92 mg/kg(100 μCi/kg) intravenous injection of stable, [^14^C]-radiolabeled CN-105 peptide(Fig. 4). Our analysis demonstrated an initial(0–4 hours post injection) plasma half-life of 29 minutes, with t_max_ in serum of 5 minutes after injection(the first time point tested). The t_max_ in the brain was 30 minutes after injection. The total CNS exposure(0–4 hours post injection) as calculated by area under the tissue concentration curve divided by area under the plasma concentration curve was 60%(Fig. 4). The progressive increase in radioactivity in the brain as compared to blood(3.6% at 5 minutes and 170% at 24 hours) demonstrates access to the CNS compartment rather than the blood in the cerebral microvasculature(Table 3). Of note, these studies were performed in uninjured animals, and it is likely that levels would be higher following forms of CNS injury that are associated with breach of the blood brain barrier.

## Discussion

In the current study we demonstrate that a rationally designed, five amino acid peptide that mimics the polar face of the receptor binding region of endogenous apoE(CN-105) improves microscopic and functional endpoints following acute traumatic brain injury. In particular, treatment with CN-105 was associated with a reduction in neuroinflammatory responses, as assessed by histological evidence of reduced microgliosis and downregulated expression of the majority of inflammatory gene mRNAs following injury. These findings were accompanied by a reduction in neuronal injury in the hippocampus, which is selectively vulnerable following trauma^44^. Treatment with CN-105 after injury was well tolerated and associated with durable functional benefits, as defined by a reduction in vestibulomotor and neurocognitive deficits. Our results are consistent with prior data demonstrating that apoE and apoE-mimetic peptides decrease neuroinflammatory responses and secondary cell death in cell culture and animal models of acute brain injury^22^,^23^,^45^,^46^,^47^,^48^.

The activity of anti-inflammatory drugs may be directly assessed through changes in the levels of pro- and anti-inflammatory mediators as well as the reduction of the number and activation state of inflammatory cells. However, cytokine mediators may work in very low concentrations, which may contribute to conflicting reports regarding level and time of activation after injury. For example, although several groups have reported that mRNA levels of inflammatory cytokines and chemokines return to pre-injury levels by 24 hours^49^,^50^,^51^, other groups report that increased levels persist for 24 hours or longer in rodents and human patients, reviewed in refs 52 and 53.

To date, a body of microglial research has been published using M1 and M2 polarization phenotypes based on macrophage terminology with the assumption that microglia mirror the function of peripheral macrophage in the brain. It is often difficult to characterize endogenous microglia(primitive macrophage entering the embryotic brain and maintained in the brain by cell division) from hematogenous macrophage that are recruited into the brain following acute injury. However, the he fact that the number and activation status(determined by morphological analysis) of microglia following injury is also decreased by treatment with CN-105 is consistent with the anti-inflammatory actions of this peptide.

Here we report that many inflammatory indicators are still greatly elevated in our TBI model at 24 hours post injury and while the upregulation of several traditional indicators of inflammation are decreased by treatment with CN-105, some such as TNFα remain elevated. It is important to note that mRNA levels cannot be used as surrogates for corresponding protein levels without verification, especially in the setting of acute brain injury. Although the clues provided as to the mechanism of TBI inflammation and the downregulation by CN-105 is exciting, this mechanistic data needs to be regarded as preliminary, and results need to be comfirmed by protein analysis.. However, recent published reports have indicated that some cytokines that have initially been thought to be detrimental in propagating the immune response, such as TNF-alpha, may actually have a dichotamous effect, exacerbating inflammation in the initial stages while aiding in recovery in the later stages^54^,^55^,^56^ and reviewed in refs 57 and 58. Furthermore, there is evidence that several of the genes that show increased expression after injury have also demonstrated increased expression in recent studies. For example, MyD88, an adaptor protein for inflammatory pathways that include signal transduction via the TLR and IL-1 receptor families, was significantly increased after experimental TBI in rodent models and in the human brain after TBI^59^,^60^. Also, Koedel and colleagues demonstrated that loss of MyD88 reduced acute brain injury and improved neurological status^61^. It is promising that our experiments show that our model of TBI increases many of these indicators of inflammation, which are reduced by treatment with CN-105.

**Figure 5:**
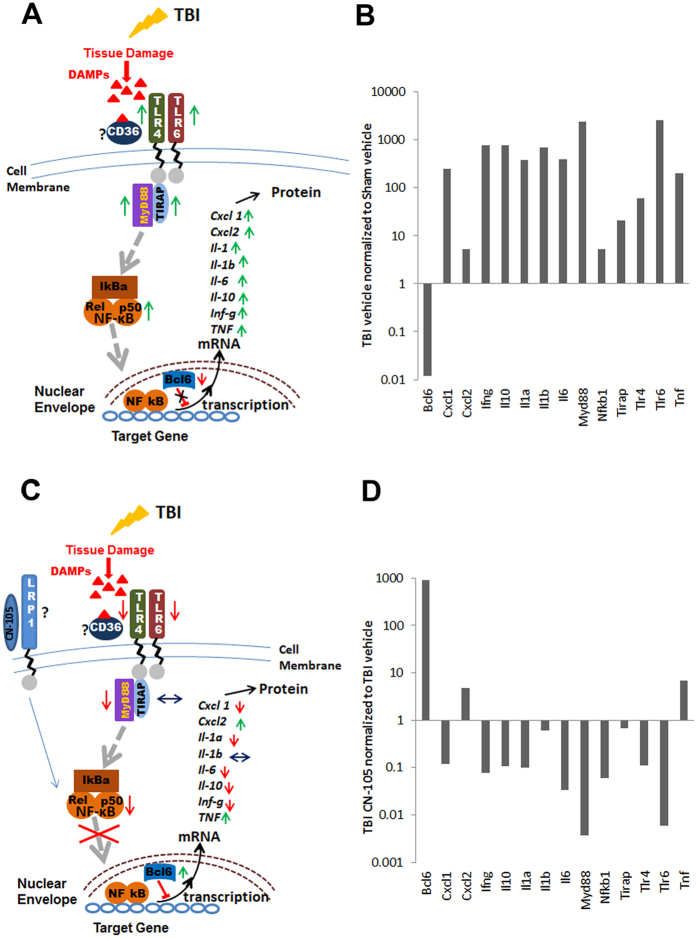
TLR signaling is downregulated by CN-105. (**A**) A schematic of the signaling pathway proposed for sterile inflammation post injury. Green arrows indicate upregulation and red arrows downregulation of genes 24 hours after TBI.(**B**) Graphic representation of genes 24 hours post TBI demonstrating fold change compared to sham control.(**C**) CN-105 modifies the post-injury inflammatory response. Arrows indicate reduced upregulation(green) and reduced downregulation(red) of gene expression when TBI injured mice are treated with CN-105 as compared to vehicle.(**D**) Graphic representation of differential gene expression 24 hours post TBI demonstrating fold change in gene expression of CN-105 treated mice normalized to vehicle treated TBI mice.

The mechanism(s) by which apoE and CN-105 are able to modulate the inflammatory response of the brain to injury remain incompletely defined. One potential mechanism by which apoE and apoE mimetic peptides may affect brain injury responses is via specific receptor interactions that initiate a signaling cascade that modify downstream expression of inflammatory genes. It has been shown that glial secretion of apoE is upregulated in the injured brain, and although there is likely redundancy between a number of apoE receptors, the LRP-1 receptor appears to play a primary role in mediating its anti-inflammatory effect in an isoform-specific fashion^62^,^63^. Moreover, apoE and apoE peptides have been demonstrated to bind the LRP receptor^32^,^64^, and interaction with the LRP receptor initiates a signaling cascade associated with the downregulation of inflammatory phenotype, an effect that is not observed in LRP-deficient microglia^7^,^33^,^34^. Moreover, apoE and apoE mimetic peptides directly reduced excitotoxic neuronal injury, an effect that is likely mediated by indirect effects of apoE receptors on the NMDA receptor complex via the cytoplasmic adaptor protein PSD-95^31^,^45^,^48^. ApoE-LRP interactions have also been associated with a decrease in the translocation of the transcription factor NF-KB which would also have the net effect of inhibiting inflammation following injury^65^. This mechanism is consistent with the differential gene expression results in the current study, which demonstrated a decrease in Nfkb1 gene expression and the expression of downstream inflammatory cytokines 24 hours post-injury. The reduction in cytokines could also result from the finding that CN-105 suppresses the upregulation of the gene for the DNA binding p50 unit of NF-κβ(*Nfkb1*)(Fig. 3) that is seen 24 hours post TBI(Fig. 5) which would be expected to further reduce the inflammatory response mediated by NF-κβ activation.

Our gene expression data suggest that the early changes in NF-kB, chemokines and cytokines may be linked to changes in BCL6 and other factors that are key in regulating the TLR mediated sterile inflammation pathway. The possibility that the apoE mimetic peptide modifies sterile inflammation in the central nervous system is particularly relevant after traumatic brain injury, where glial activation and neuroinflammatory responses exacerbate secondary tissue injury. Toll like receptors(TLRs) have been found to have a role in models of sterile inflammation(reviewed in refs 66 and 67)^67^,^68^,^69^. TLR4 and TLR6 complexed with the scavenger receptor CD36 has been shown to signal through the intracellular adapter, *MyD88*, and *NF-KB* to stimulate chemokine(*Cxcl1 and Cxcl2*) and cytokine(*IL-1a, IL-6, IL-10, Inf-γ and TNF-α*) expression^70^. Here we report that twenty-four hours after TBI, many genes in this signaling pathway are upregulated(Fig. 5A and B, Table 1) with *Myd88* and *TLR6* showing the highest level of upregulation, and TLR4, IL-1a, IL-6, IL-10 and Cxcl1 demonstrating a moderate level of upregulation. In addition, it has been recently discovered that the leukemia oncogene Bcl6 is the master regulator of many crucial pathways including NF-KB signaling related inflammation^71^,^72^,^73^. As predicted by the published data, the repression of the gene for Bcl6 by TBI in our model coincides with increased levels of transcription of several TLR genes, *Myd88, CD40, INFγ, IL-1a, IL-10, IL-6*(Fig. 5A and B) along with the upregulation of *BCL6* mRNA the majority of those same genes is repressed in TBI mice treated with CN-105(Fig. 5C and D). Thus, it is plausible that CN-105 modifies the acute post-injury neuroinflammatory response by interaction with LRP1, resulting in reduction of inflammatory genes mediated by the NF-KB and BCL-6 regulated pathways and thus ultimately reducing TLR mediated sterile inflammation and mitigating secondary neuronal injury, resulting in durable functional benefit. It is also possible that CN-105 has a direct effect on blood brain barrier integrity. This is consistent with recent results demonstrating that CN-105 reduced cerebral edema in a murine model of intracranial hemorrhage^74^, and that apolipoprotein E may directly effect the blood brain barrier through regulation of tight junctions^63^,^75^.

There are a number of challenges associated with the translation of a new therapeutic strategy to the clinical setting. One advantage of a therapeutic strategy that modifies post-traumatic neuroinflammatory responses is the potential for a relatively long therapeutic window, as glial activation and the development of cerebral edema peaks during the first several days of injury. In this regard, defining the post-injury latency during which a therapeutic intervention retains its beneficial effect is an important variable in informing a pilot clinical trial. In the current trial, we found that pre-treatment with CN-105 was not necessary. In fact, functional improvement was retained when peptide was administered up to 4 hours following injury. In further studies, this temporal window should be further extended.

Several limitations to the current study should be addressed. For example, apoE-based peptides derived from the receptor binding region of apoE are not an ideal model to study the isoform specific effects of the endogenous holoprotein, as the amino acid substitutions that define the common APOE polymorphisms flank the receptor binding region, and likely modulate receptor binding via allosteric effects^76^. Another potential limitation in these results is that, although the differential gene expression results are consistent with the histological features of reduced microgliosis and CNS inflammation as a function of CN-105 administered post-injury, it should also be noted that this conclusion may be biased by the fact that the differential gene assay was focused on expression of inflammatory mediators.

Given the failure of multiple neuroprotective trials, the translatability of rodent models to clinical TBI remains an area of debate. For example, there are a number of limitations inherent to all rodent models of traumatic brain injury. This includes the lissencephalic nature of rodent brains, which precludes testing of higher and more subtle cortical functions that are directly applicable to the clinical setting. Moreoever, rodent brains have reduced ratio of white: grey matter as compared to primates, and thus are not ideal to model the diffuse axonal injury that may occur in the clinical setting. Murine TBI models also largely fail to recapitulate the biomechanical forces transmitted to human brains. Despite these limitations, a number of murine models have been developed to model focal injury(controlled cortical impact models) and closed head injury. It is important to note that all of these models have potential limitations and advantages, and may provide complementary information(reviewed in refs 77 and 78). Our model of closed head injury was chosen because it replicates many of the histological features(diffuse gliosis, hippocampal injury) and clinical sequelae(vestibulomotor deficits and long term neurocognitive deficits) associated with human moderate closed head injury. As noted above, although all rodent TBI models have relative advantages and liabilities, we have recently demonstrated that CN-105 reduces cerebral edema and improves functional outcome in a model of intracranial hemorrhage^74^. This supports recent observations from our laboratory and others that apoE based peptides improve other facets of TBI pathology, including cerebral ischemia^79^, intraparenchymal hemorrhage^74^, and subarachnoid hemorrhage^25^,^26^. An additional consideration is that, although an overly robust neuroinflammatory response may exacerbate tissue injury in the acute setting, there is evidence that activated glia may play an adaptive role in the subacute setting by providing trophic support and mediating adapative synaptic reorganization^80^,^81^. Finally, although peptide-based therapeutics may be appropriate for acute brain injury, they are likely to have limited oral bioavailability, and small molecule mimetics would likely be more appropriate for chronic neurodegenerative conditions associated with CNS inflammation.

In conclusion, we find that treatment with a small five amino acid residue peptide derived from the receptor binding region of apoE improved functional outcomes following closed head injury. These functional improvements in vestibulomotor and neurocognitive behavior were durable throughout the 31 day testing period, and were retained even when the first administration of apoE peptide was withheld until 4 hours following TBI. Moreover, administration of CN-105peptide was well tolerated, and these functional improvements were associated with a reduction in hippocampal neuronal injury, microgliosis, and inflammatory gene expression. The further development of apoE based therapeutics represents a promising therapeutic strategy in the treatment of acute brain injury.

## Methods

This study was carried out in strict accordance with the recommendations in the Guide for the Care and Use of Laboratory Animals of the National Institutes of Health. The protocol was approved by the Duke University Institutional Animal Care and Use Committee, Durham North Carolina, protocol #: A030-12-02. All surgery was performed under isoflurane anesthesia.

### Closed Head Injury Model

The murine closed head injury model used in this study was previously described^29^. The closed head impact results in injury to selectively vulnerable neurons in cortex and hippocampus, and is associated with vestibulomotor deficits and long term neurocognitive deficits. Although animals do lose body weight, they rapidly regain spontaneous ventilation, righting reflex, and the ability to ambulate. Briefly, 12–14 week-old C57Bl/6 J male mice(Jackson Laboratories, Bar Harbor, ME) were used. The trachea was intubated after anesthesia induction with 4.6% isoflurane and the lungs were mechanically ventilated with 1.6% isoflurane in 30% O2/70% N2. Core body temperature was maintained at 37 °C through a rectal probe. To avoid basilar skull fracture, ear bars are not used. The animal was secured in a stereotactic device in a prone position on a molded acrylic cast with surgical tape across the shoulders. The intubation tube was also secured to the the acrylic cast with tape. The acrylic cast is designed to allow the mouse to have 3 mm of space below the head to allow for acceleration/decelaration of the head in this position. The head was shaved and the scalp was incised to expose the skull and identify anatomical landmarks. A concave 3-mm metallic disc was adhered to the skull immediately caudal to bregma. A 2.0-mm diameter pneumatic impactor(Air-Power Inc., High Point, NC) was used to deliver a single midline impact to the center of the disc surface. The impactor was discharged at 6.8 ± 0.2 m/second with a head displacement of 3 mm. After impact, the animals were allowed to recover spontaneous ventilation and then the tracheas were extubated. Mice were allowed free access to food and water. Sham mice were treated identically except for the absence of impact. All mice were housed in the same facility.

### Drug Administration

CN-105(Ac-VSRRR-amide) was synthesized by Polypeptide Inc.(San Diego, CA) to a purity of >99%, and was delivered in sterile normal saline. Animals were placed in a restrainer(Harvard Apparatus, Holliston, MA), and a single intravenous dose of drug(0.05 mg/kg) was administered by tail vein in a volume of 100 μL. Vehicle treated animals received intravenous injection of 100 μL of normal saline at the same time points. Animals were assigned to treatment group by a coded study identification number after injury using a paper randomization protocol. A block randomization scheme was used, so that an equal number of animals were randomized to each of the treatment groups during concurrent experiments.

### Immunohistochemistry

To assess the effects of CN-105 on inflammation, neuronal injury and neuronal loss, immunohistochemical(IHC) staining was performed using the F4/80 antibody(a marker for mature microglia and macrophages; rat monoclonal, 1:10,000; Serotec, Raleigh, NC) and the Fluoro-Jade B stain(a marker of degenerating neurons; Histo-Chem Inc. Jefferson, AR) on days 10 and 1, respectively, after TBI. IHC was performed on separate cohorts of mice from those used in neurobehavioral tests. As previously described^82^, mice were anesthetized, euthanized, and perfused with 30 ml phosphate-buffered saline(PBS) via transcardiac puncture. The brains were then immersed in buffered formalin overnight and saturated with 30% sucrose in buffer. Sagittal sections(40 μm) were sliced on a freezing microtome and collected in cryoprotectant solution. For histological assessment the following were use: Secondary antibody, biotinylated goat anti-mouse IgG(1:3,000), ABC, and DAB all from Vector Laboratories, Inc., Burlingame, CA. and Gill’s Hematoxylin, Fisher Scientific, Fair Lawn, NJ.

### Cell Quantification and Image Analysis

Prior to quantification, all slides were coded and the analyst was blinded to avoid experimenter bias. For the F4/80 quantification the brains of 5 TBI treated and 6 TBI vehicle treated mice(4–6 sections/mouse) were counted. Every eighth section of the dorsal hippocampus, according to the Paxinos and Franklin(2001) mouse brain atlas, was analyzed using the Stereo Investigator 7.0 software(MicroBrightField, Williston, VT). The entire hippocampus was outlined using a 4x objective. Immunopositive microglia with visible nuclei were identified with a 20x objective, and the total numbers were estimated with the optical fractionator method(West *et al*., 1991). An average of 12 counting frames was analyzed per section, the grid size was 450 × 450 μm, and the dissector dimensions were 80 × 80 × 10 μm. As we did not quantify through the entire hippocampus, results are presented as objects per unit volume(mm^3^) to allow comparability between animals(number of objects estimated/total volume evaluated). Group averages for vehicle and CN-105 treated animals were generated and compared using the Student’s *t* test function(GraphPad Prism software).

For Fluoro-Jade B, brain sections from 12 TBI mice(6 vehicle and 6 CN-105) containing the dorsal hippocampus(5–6 sections per animal) were examined for degenerating neurons using an epifluorescence microscope(Nikon, Tokyo, Japan) with a medium band blue excitation(Nikon B-2A, 450–490 nm) filter set. Images of the dentate gyrus were acquired and a virtual grid was placed over the image. Degenerating neurons were counted and the total number of Fluoro-Jade B-positive neurons per every eighth brain slice was recorded. Group averages for vehicle and CN-105 treated animals were generated and compared using the Student’s *t* test function(GraphPad Prism software).

### Testing of Functional Deficits

Mice were randomly assigned to treatment groups immediately following injury and all behavioral evaluations were performed by investigators blinded to treatment.

An automated Rotarod(Ugo Basile, Comerio, Italy) was used to assess vestibulomotor function^83^. On the day prior to injury, mice(n = 11–12 mice per group) underwent one training trial at an accelerating rotational speed(4–40 rpm) for at least 200 seconds and then three additional test trials with the same accelerating rotational speed. The average time to fall from the rotating cylinder in the test trials was recorded as baseline latency. Mice were tested on consecutive days post-injury and received three consecutive daily trials with accelerating rotational speed(inter-trial interval = 15 minutes)(n = 10–12 mice). The average latency to fall from the rod was recorded. Mice unable to grasp the rotating rod were given a latency value of 0 seconds.

As described previously the Morris Water Maze assesses spatial learning and memory by testing the ability of mice to locate a submerged platform^36^. The mice were placed in a pool(105 cm diameter) filled with room temperature(25 °C) water made opaque with fat free powdered milk and allowed up to 90 seconds to locate the submerged platform. The mice performed four trials/day for 4 consecutive days(inter-trial interval = 30 min). The mice were introduced in varying quadrants of the pool for each trial but the location of the platform never varied. The latency to locate the platform was recorded, and the 4 trials per day were averaged. Mice were tested on days 28–31 post-injury(n = 11–12 mice per group). A probe trial was administered on day 4 of the experiment 3 hours after the last test trial was completed. For the probe trial, the platform was removed and the mice were allowed to swim freely for 60 seconds. The percent of the time the mice spent in the platform quadrant was quantified.

To ensure that vision was intact in TBI injured mice a separate cohort of 6 TBI and 6 sham mice was assessed. On day 28 a visible platform with a flag attached to a wire that extended above the platform was placed in the pool and mice were introduced in varying quadrants of the pool for 4 trials, the latency was recorded. An hour following the visible trial the flag was removed from the platform and the platform was placed in a new location and four new trials were performed and the latency was recorded. The four trials were averaged.

### RNA extraction and RT-PCR

Frozen, pulverized whole brain tissue was processed for RNA extraction from a separate cohort of treated and untreated injured animals on days 1 post-injury(CN-105, n = 4; vehicle, n = 3; Control(sham-operated), n = 3). Total RNA was extracted from frozen, pulverized whole brain tissue using RNeasy Lipid Tissue Mini Kit(Qiagen, Valencia, CA). RNA quantity and quality was assessed with the NanoDrop ND-1000 spectrophotometer(NanoDrop Technologies, Inc., Wilmington, DE) and by agarose gel electrophoresis. Only samples with a 260/280 ratio between 1.9–2.1, and a 260/230 ratio greater than 2.0, were further processed. First strand complementary DNA(cDNA) was generated from 2 μg total RNA using the RT^2^ First strand kit(SABiosciences, Frederick, MD.)

Gene expression was measured using the Mouse Inflammatory Response and Autoimmunity PCR Array(SABiosciences, Frederick, MD), which profiles the expression of 84 genes related to inflammatory and autoimmune processes. RT-PCR was performed according to manufacturer’s instructions Quality of the cDNA and PCR efficiency was verified by housekeeping genes and RT-PCR controls included in the PCR Array.

### Gene expression data analysis

Raw RT-PCR data were analyzed using the Web-Based PCR Array Data Analysis software(SABiosciences). ΔC_t_ values and ΔΔC_t_ - based fold-change were calculated from raw threshold cycle data, using beta-actin and glyceraldehyde 3-phosphate dehydrogenase(GAPDH) as internal standards for normalization. Fold changes were then normalized against sham-operated controls.

### Pharmacokinetic sample preparation and analysis

Pharmacokinetic studies were conducted by Xenobiotic Laboratories(Plainsboro, NJ). Briefly, male CD-1 mice received a single IV bolus dose of [^14^C] CN-105 52 μCi/mg(dose of 1.92 mg/kg, and approximately 100 μCi/kg) by tail vein injection. At 0.083, 0.5, 1, 6, 24, and 48 hours post-dose terminal blood samples were collected from 2 animals per timepoint and plasma was isolated for radiometric analysis.

To assess CNS penetration, quantitative whole body autoradiography(QWBA) analysis was performed and areas of interest in blood and brain were compared. The carcasses were frozen in a dry ice/hexane bath and embedded in low viscosity carboxymethylcellulose for sagittal, 30-μm thick sections of low viscosity carboxymethylcellulose-of mouse carcasses were sectioned. Pharmacokinetic data were generated by non-compartmental PK analysis of total radioactivity concentration vs. time profiles for tissues using WinNonlin(version 6.3, Pharsight Corporation, Mountain View, CA).

Selected tissues and areas of interest(AOI) were analyzed using QWBA. The tissues and AOI were analyzed within AIDA software using a region sampling tool. The following parameters were derived for individual subjects: maximum tissue and plasma concentration(C_max_), time of C_max_(t_max_), area under the tissue concentration versus time curve from time zero to the last quantifiable tissue concentration(AUC_0-t_), area under the tissue concentration versus time curve from time zero to 4 hours(AUC_0-4_), and the apparent terminal half-life(t_½_). AUC_0-4_ was calculated using the linear trapezoidal rule. Results for AOIs are expressed in nanogram equivalents of(^14^C) CN-105 per gram of tissue(ng equv./g) and were calculated as follows: ng equiv./g is equal to nCi per gram of tissue per nCi/ng of(^14^C) CN-105.

### Statistical analysis

After testing for normality of data by constructing normal probability plots with raw data, serial tests of functional performance, including Rotarod and MWM performance, were compared with a two factor repeated measures analysis of variance(ANOVA) with time as the repeated variable. When F-value was significant for group effect, pairwise comparison was performed using *post-hoc* Scheffe test for correcting multiple comparisons. The number of F4/80 and Fluoro-Jade B positive cells was compared among groups with the Kruskal–Wallis H statistic. Between groups, differences were compared by the Mann–Whitney U statistic. Parametric values are expressed as mean ± standard deviation(SD). Significance was assumed if p < 0.05.

## Additional Information

**How to cite this article:** Laskowitz, D. T. *et al*. Neuroprotective pentapeptide CN-105 is associated with reduced sterile inflammation and improved functional outcomes in a traumatic brain injury murine model. *Sci. Rep.* **7**, 46461; doi: 10.1038/srep46461(2017).

**Publisher's note:** Springer Nature remains neutral with regard to jurisdictional claims in published maps and institutional affiliations.

## Figures and Tables

**Table 1:** Inflammatory gene expression, ΔC_t_ values and fold change relative to Sham-vehicle.

Gene	S-v	S-CN-105	TBI-v	TBI-CN-105	S-CN-105/S-v	TBI-v/S-v	TBI-CN-105/S-v
Cxcl3	3.0E − 05	2.3E − 05	6.7E − 01	1.6E + 00	0.8	22171.7	54032.0
Ccl11	7.8E − 05	2.5E − 04	6.4E − 01	1.8E − 01	3.2	8199.7	2253.1
Ccr4	3.9E − 06	6.0E − 06	2.6E − 02	3.1E − 02	1.5	6537.5	7945.9
Ccl20	2.6E − 05	1.1E − 04	1.2E − 01	7.9E − 02	4.4	4574.0	3086.8
Tlr6	4.7E − 04	6.2E − 04	1.2E + 00	7.0E − 03	1.3	2528.8	14.9
Ccl24	3.2E − 05	9.7E − 05	7.6E − 02	5.1E − 03	3.0	2356.9	159.1
Myd88	7.8E − 04	1.1E − 03	1.8E + 00	6.6E − 03	1.5	2327.8	8.5
Cxcl9	6.8E − 05	1.0E − 04	1.5E − 01	2.2E − 03	1.5	2265.1	32.2
Lta	1.2E − 04	2.6E − 04	1.3E − 01	1.1E − 01	2.3	1133.5	917.0
Tnfsf14	2.5E − 05	1.3E − 04	2.5E − 02	9.0E − 03	5.2	1027.7	365.9
Il9	1.2E − 05	*	1.2E − 02	1.4E − 02	**	957.7	1155.5
Ifng	7.3E − 05	5.8E − 05	5.6E − 02	4.3E − 03	0.8	767.1	59.7
Il10	1.9E − 05	1.1E − 04	1.5E − 02	1.6E − 03	5.7	766.1	82.2
Il1b	1.1E − 03	2.0E − 03	7.5E − 01	4.6E − 01	1.8	683.8	416.6
Il8rb	8.3E − 06	6.1E − 05	5.2E − 03	5.4E − 03	7.3	623.4	658.4
Il6	1.4E − 04	1.1E − 04	5.4E − 02	1.8E − 03	−1.3	395.1	13.2
Il1a	1.4E − 03	1.9E − 03	5.5E − 01	5.4E − 02	1.4	382.3	37.7
Cd40	5.4E − 04	8.3E − 04	1.6E − 01	2.1E − 02	1.5	301.4	38.8
Ccr2	2.6E − 04	5.5E − 04	7.1E − 02	1.5E − 03	2.1	271.6	5.6
Cxcl1	6.0E − 05	1.6E − 04	1.5E − 02	1.8E − 03	2.7	244.8	29.4
Ccr7	1.2E − 04	2.1E − 04	2.7E − 02	3.4E − 02	1.8	228.3	283.2
Il18rap	1.1E − 04	4.5E − 05	2.4E − 02	6.6E − 03	0.4	211.3	58.6
Tnf	2.8E − 05	3.5E − 05	5.5E − 03	3.8E − 02	1.3	197.3	1367.7
C4b	3.6E − 03	7.0E − 03	6.3E − 01	5.9E − 01	2.0	176.5	163.3
Il8ra	1.3E − 05	*	2.2E − 03	2.6E − 03	**	168.5	196.8
Cxcr4	4.3E − 03	6.6E − 03	6.6E − 01	9.0E − 02	1.5	153.8	21.0
Cxcl11	2.4E − 05	9.0E − 05	3.4E − 03	8.8E − 03	3.8	141.3	372.3
Il6ra	3.4E − 03	4.9E − 03	4.8E − 01	1.6E − 01	1.5	140.3	47.9
Il22	4.3E − 05	4.7E − 04	5.6E − 03	3.4E − 03	10.8	129.4	77.8
Il1rn	2.3E − 05	*	2.8E − 03	1.7E − 03	**	121.0	74.2
Ccl2	5.8E − 04	8.3E − 04	6.5E − 02	2.5E − 02	1.4	110.8	42.3
C3ar1	3.5E − 03	4.1E − 03	3.7E − 01	1.2E − 01	1.2	108.2	34.9
Il1f10	1.6E − 05	*	1.5E − 03	5.5E − 03	**	93.4	352.3
Ccl4	3.7E − 04	3.8E − 04	3.0E − 02	1.2E − 02	1.0	83.4	32.3
Tlr4	3.1E − 03	3.5E − 03	1.8E − 01	2.0E − 02	1.1	59.2	6.5
Il23a	2.7E − 05	8.0E − 05	1.2E − 03	2.3E − 02	3.0	45.4	838.4
Ripk2	7.0E − 03	1.1E − 02	2.3E − 01	2.1E − 01	1.5	32.0	30.1
Tlr7	1.7E − 03	2.6E − 03	4.8E − 02	9.1E − 01	1.6	28.7	546.2
Cxcl10	9.2E − 04	1.9E − 03	2.5E − 02	5.3E − 04	2.1	27.6	−1.7
Tlr1	4.8E − 04	1.0E − 03	1.1E − 02	7.4E − 03	2.2	22.0	15.4
Tirap	3.8E − 03	7.8E − 03	7.9E − 02	5.3E − 02	2.1	21.0	14.0
Ltb	5.1E − 04	1.0E − 03	1.1E − 02	3.9E − 02	2.0	20.9	75.7
Ccl25	5.6E − 03	1.4E − 02	1.1E − 01	9.5E − 02	2.6	20.3	16.9
Hdac4	6.0E − 02	9.1E − 02	9.0E − 01	8.3E − 01	1.5	14.9	13.9
Itgb2	5.5E − 03	1.3E − 02	8.1E − 02	5.3E − 02	2.5	14.7	9.6
Ccr3	1.7E − 03	2.1E − 03	2.1E − 02	1.1E − 01	1.2	12.3	66.6
Ccl3	5.1E − 04	8.0E − 04	6.1E − 03	8.9E − 03	1.6	12.0	17.7
Il7	1.1E − 04	3.2E − 04	1.1E − 03	5.9E − 01	3.1	10.8	5569.8
Tlr2	9.1E − 04	1.3E − 03	9.0E − 03	9.1E − 03	1.4	9.9	10.0
Fasl	2.1E − 04	1.9E − 04	1.3E − 03	6.7E − 04	0.9	6.3	3.2
Ccl5	8.9E − 04	1.1E − 03	5.5E − 03	3.4E − 03	1.2	6.2	3.8
Cxcl2	6.6E − 05	2.8E − 04	3.5E − 04	1.7E − 03	4.2	5.3	25.5
Nfkb1	1.7E − 02	3.1E − 02	8.8E − 02	5.3E − 03	1.8	5.3	−3.1
Cebpb	6.8E − 02	1.1E − 01	3.5E − 01	2.4E − 01	1.6	5.2	3.5
Ccl7	1.3E − 03	1.6E − 03	4.4E − 03	3.7E − 03	1.3	3.5	2.9
C3	4.1E − 04	7.0E − 04	1.3E − 03	1.9E − 03	1.7	3.1	4.6
Tlr3	1.5E − 02	2.3E − 02	4.5E − 02	5.8E − 02	1.6	3.0	3.9
Ccl22	1.2E − 04	3.8E − 04	3.6E − 04	2.2E − 02	3.1	2.9	176.2
Csf1	1.1E − 02	1.9E − 02	2.4E − 02	1.2E − 02	1.7	2.3	1.1
Fos	1.5E − 01	2.3E − 01	1.9E − 01	1.7E − 01	1.6	1.3	1.1
Tlr5	6.8E − 04	6.3E − 04	6.6E − 04	1.7E − 03	0.9	1.0	2.5
Il23r	1.1E − 04	1.5E − 04	1.4E − 04	8.0E − 04	1.4	−0.8	7.2
Tollip	1.6E − 01	3.2E − 01	1.5E − 01	5.1E − 02	1.9	−1.1	−3.2
Ccl19	1.9E − 02	2.9E − 02	1.7E − 02	3.4E − 03	1.5	−1.1	−5.8
Ccl17	2.6E − 03	4.2E − 03	1.7E − 03	1.0E − 03	1.6	−1.5	−2.6
Il10rb	1.5E − 02	3.0E − 02	8.4E − 03	2.2E − 02	2.0	−1.8	1.5
Flt3l	2.0E − 03	3.0E − 03	9.6E − 04	7.1E − 04	1.5	−2.1	−2.8
Ly96	3.8E − 03	7.2E − 03	1.2E − 03	7.7E − 02	1.9	−3.1	19.9
Il22ra2	2.4E − 05	1.1E − 04	7.5E − 06	8.2E − 02	4.8	−3.2	3448.3
Ccl8	7.5E − 04	2.1E − 03	1.3E − 04	4.2E − 03	2.8	−5.6	5.6
Ccr1	7.5E − 04	7.1E − 04	1.3E − 04	3.0E − 04	−1.1	−5.8	−2.5
Cxcl5	1.4E − 03	2.5E − 03	2.2E − 04	1.4E + 00	1.7	−6.5	957.4
Il1rap	3.1E − 02	4.4E − 02	1.8E − 03	8.2E − 04	1.5	−17.1	−37.5
Ccl12	3.6E − 03	6.5E − 03	2.1E − 04	1.3E − 02	1.8	−17.3	3.7
Il1r1	4.6E − 03	1.2E − 02	2.2E − 04	7.5E − 04	2.6	−21.5	−6.2
Nfatc3	8.7E − 03	2.1E − 02	3.2E − 04	3.2E + 00	2.4	−27.5	364.7
Il18	9.1E − 02	1.8E − 01	1.3E − 03	9.6E − 03	2.0	−68.6	−9.5
Bcl6	5.1E − 02	6.6E − 02	6.2E − 04	5.7E − 01	1.3	−82.1	11.2
Nr3c1	2.1E − 01	4.0E − 01	8.5E − 04	1.2E − 02	2.0	−243.3	−17.8
Ccl1	*	2.3E − 05	6.3E − 01	9.1E − 01	**	**	**
Cd40lg	*	6.9E − 05	4.9E − 03	4.2E − 03	**	**	**
Crp	*	*	5.9E − 04	7.1E − 04	**	**	**
Kng1	*	1.4E − 05	9.2E − 03	8.5E − 03	**	**	**
Nos2	8.8E − 05	8.9E − 05	*	1.6E − 01	1.0	**	1779.4

Inflammatory gene expression 24 hours post sham or TBI injury. Data is organized by fold change of TBI-vehicle normalized to sham-vehicle.

*Indicate mRNA expression was below the level of detection.

**Indicate relative value could not be calculated because one of the components was below the level of detection.

**Table 2:** Inflammatory gene expression of CN-105 relative to vehicle treated group following TBI.

Gene	Fold Change	Gene	Fold Change	Gene	Fold Change
*Il22ra2*	10895	Tlr3	1.3	*Fasl*	−2.0
*Nfatc3*	10044	Ccr7	1.2	*Csf1*	−2.0
*Cxcl5*	6257	Ccr4	1.2	*Il1rap*	−2.2
*Bcl6*	921	Il9	1.2	*Ccl4*	−2.6
*Il7*	515	Crp	1.2	*Ccl2*	−2.6
*Ccl12*	64.2	Il8ra	1.2	*Tnfsf14*	−2.8
*Ly96*	61.2	Il8rb	1.1	*Tollip*	−2.9
*Ccl22*	60.2	Tlr2	1.0	*Il6ra*	−2.9
*Ccl8*	31.2	Ripk2	−1.1	*C3ar1*	−3.1
*Tlr7*	19.0	Kng1	−1.1	*Il18rap*	−3.6
*Il23a*	18.5	Hdac4	−1.1	*Ccl11*	−3.6
*Nr3c1*	13.7	C4b	−1.1	*Ccl19*	−5.2
*Il18*	7.2	Fos	−1.2	*Cxcr4*	−7.3
*Tnf*	6.9	Cd40lg	−1.2	*Cd40*	−7.8
*Il23r*	5.8	Ccl7	−1.2	*Cxcl1*	−8.3
*Ccr3*	5.4	Ccl25	−1.2	*Tlr4*	−9.0
*Cxcl2*	4.8	Lta	−1.2	*Il10*	−9.3
*Il1f10*	3.8	Flt3l	−1.4	*Il1a*	−10.1
*Ltb*	3.6	Tlr1	−1.4	*Ifng*	−12.9
*Il1r1*	3.5	Ccl20	−1.5	*Ccl24*	−14.8
*Cxcl11*	2.6	Cebpb	−1.5	*Nfkb1*	−16.6
*Il10rb*	2.6	Tirap	−1.5	*Il6*	−29.8
*Tlr5*	2.5	Itgb2	−1.5	*Cxcl10*	−47.9
*Cxcl3*	2.4	Il1rn	−1.6	*Ccr2*	−48.3
*Ccr1*	2.3	Ccl5	−1.6	*Cxcl9*	−70.3
*C3*	1.5	Il1b	−1.6	*Tlr6*	−170
*Ccl3*	1.5	Il22	−1.7	*Myd88*	−273
*Ccl1*	1.5	Ccl17	−1.7	*Nos2*	*

Fold Change-Inflammatory gene expression 24 hours post TBI with CN-105 treatment relative to vehicle. Greater than 3 fold change-light gray, unchanged-unshaded, below threshold of detection dark-gray.

*Indicate mRNA expression was below the level of detection.

**Table 3:** Percent of radioactivity contributed to blood in the brain microvasculature.

	Radioactivity(ng equiv/g) in tissue following bolus(hours = h)					
	0.08 h	0.5 h	1 h	6 h	24 h	48 h
*Blood(Cardiac*)	1102	500	95	15	14.1	15.5
*Brain*	39.5	71.2	32.5	24.9	24.1	20.9
*% Brain*/*Blood*	3.58%	14%	34%	167%	170%	135%
